# Walking Capacity and Daily Movement Behaviours After Ischemic Stroke: A Potential Capacity‐Performance Gap

**DOI:** 10.1002/pri.70372

**Published:** 2026-11-03

**Authors:** Joy C. Ezeugwa, Mona Iyizoba, Asya Shiloff‐Rogers, Andrew Chan, Jake Hayward, Carmen Tuchak, Jaime C. Yu, Brian H. Buck, Patricia J. Manns, Victor E. Ezeugwu

**Affiliations:** ^1^ Department of Physical Therapy Faculty of Rehabilitation Medicine University of Alberta Edmonton Alberta Canada; ^2^ Glenrose Rehabilitation Hospital Edmonton Alberta Canada; ^3^ Department of Emergency Medicine Faculty of Medicine and Dentistry University of Alberta Edmonton Alberta Canada; ^4^ Division of Physical Medicine and Rehabilitation Faculty of Medicine and Dentistry Department of Medicine University of Alberta Edmonton Alberta Canada; ^5^ Division of Neurology Faculty of Medicine and Dentistry University of Alberta Edmonton Alberta Canada

**Keywords:** accelerometry, gait speed, physical activity, sedentary behaviour, stroke rehabilitation, walking capacity, wearable sensors

## Abstract

**Background and Purpose:**

Walking capacity is a common focus of stroke rehabilitation; however, the extent to which walking capacity is associated with daily movement behaviours (primary lying, sedentary time, standing and stepping) during different phases of stroke recovery remains unclear. This study examined the relationship between walking capacity and daily movement behaviours among individuals in the acute and subacute phases of ischemic stroke recovery.

**Methods:**

In this cross‐sectional study, adults with ischemic stroke who were ambulatory (able to walk ≥ 5 m with or without a gait aid) were recruited during the acute (≤ 7 days post‐stroke; *n* = 19) and subacute (2 weeks‐3 months post‐stroke; *n* = 25) phases of stroke recovery. Movement behaviours were assessed using 24‐h activPAL accelerometry, including primary lying time, sedentary time, standing time, stepping time and daily step count. Walking capacity was assessed using the 6‐Minute Walk Test, 10‐m Walk Test (gait speed) and Timed Up and Go. Between‐group differences were analysed using Welch’s *t*‐tests or Mann‐Whitney U tests, as appropriate, and associations were examined using Spearman correlations and exploratory partial correlations adjusted for stroke severity (NIHSS).

**Results:**

The subacute group demonstrated faster gait speed than the acute group (0.89 vs. 0.71 m/s; *p* = 0.03), and shorter Timed Up and Go times (median 15.9 vs. 19.0 seconds; *p* = 0.03), whereas the 6‐Minute Walk Test and daily movement behaviours were similar. Both groups accumulated high sedentary time (∼12 hours/day). Gait speed was the only walking capacity measure associated with daily movement behaviours. In exploratory analyses adjusted for stroke severity, gait speed remained associated with sedentary time in both groups, whereas associations with stepping and standing time were attenuated in the acute group.

**Conclusions:**

In this ambulatory sample of adults in the acute and subacute phases of ischemic stroke recovery, participants accumulated approximately 12 hours of sedentary time per day despite walking speeds approaching thresholds associated with community ambulation. These findings are consistent with a potential capacity‐performance gap and highlight the importance of assessing daily movement behaviours alongside standardized walking capacity measures.

## 1 Introduction

Walking, classified within the International Classification of Functioning, Disability and Health (ICF) under the activities and participation domain, is one of the most frequently stated rehabilitation goals following stroke (Pollock et al. 2014). For most people with stroke, regaining the ability to walk independently represents a critical milestone towards autonomy and community reintegration (Fulk et al. 2017). Walking capacity is also an important factor in determining whether a patient can return home or transition to an institutionalized facility (Covert et al. 2020). Within the ICF, walking recovery can be understood through two distinct but complementary constructs: *capacity*, defined as an individual’s highest probable level of functioning in a standardized clinical environment, and *performance*, defined as what an individual actually does in their environment (World Health Organization 2022). This distinction is essential, as higher walking capacity is not consistently associated with higher walking performance (Lang et al. 2023; Lundquist et al. 2022; Thompson et al. 2024; Waddell et al. 2017). This capacity‐performance gap is well‐documented in upper limb (UL) recovery (Lang et al. 2023; Lundquist et al. 2022; Waddell et al. 2017), where gains in UL capacity (e.g., Action Research Arm Test scores) often do not result in increased paretic limb use in daily life when measured objectively via accelerometry, and emerging evidence suggests a similar disconnect in walking rehabilitation, where gains in walking capacity were not consistently accompanied by corresponding gains in daily stepping (Lang et al. 2023). Whether this potential disconnect extends to broader whole‐day movement behaviours after stroke‐including sedentary time and standing, in addition to stepping‐remains less clear. While stroke rehabilitation prioritizes improving walking capacity using measures such as the 6‐Minute Walk Test (6MWT), gait speed, and the Timed Up and Go (TUG) test (Boyne et al. 2023; Klassen et al. 2020; Moore et al. 2018), these measures may not reflect how individuals perform in their day‐to‐day lives. Wearable activity sensors provide continuous, unsupervised, and objective monitoring of movement behaviours, complementing clinical measures by capturing movement behaviour patterns that may influence long‐term functioning (Wondergem et al. 2022). In particular, daily step count provides an intuitive and clinically meaningful measure of walking performance that is not captured by traditional clinical metrics (Ding et al. 2025; Lynch et al. 2018). As outlined in the Canadian 24‐Hour Movement Guidelines (Ross et al. 2020), movement behaviours are compositionally linked: changes in time spent in one behaviour are mirrored by changes in another. For example, an increase in sedentary time may be mirrored by a corresponding decrease in light‐intensity physical activity, or vice versa. Stroke guidelines recommend at least 20 minutes or more of moderate‐to‐vigorous physical activity (MVPA; MacKay‐Lyons et al. 2020). Despite these recommendations, studies have shown that people with stroke are inactive (Ezeugwu and Manns 2017; Yan et al. 2025). Research shows that 69%–94% of waking hours are spent sedentary, with negligible time spent in MVPA post‐stroke (Barrett et al. 2018; English et al. 2016; Ezeugwu and Manns 2018; Mattlage et al. 2015). For example, within the first 6 months post‐stroke, when recovery should be at its fastest, one study reported that sedentary time was approximately 75% of waking hours, with MVPA averaging only 1.1 minutes (Ezeugwu and Manns 2017). How movement behaviours are accumulated has important clinical implications (Ezeugwu and Manns 2024; Wondergem et al. 2022). For example, prolonged sedentary time is associated with adverse cardiometabolic outcomes and higher cardiovascular mortality (Buffey et al. 2022; Pinto et al. 2023; Wang et al. 2022). Given the elevated risk of recurrent stroke, prolonged sedentary time and physical inactivity may further increase the risk and negatively impact physical functioning and long‐term recovery (Ashizawa et al. 2024; Wang et al. 2022).

The acute (within 7 days post‐stroke) and subacute (2 weeks–3 months post‐stroke) phases of recovery represent a critical period for neuroplasticity and enhanced recovery (Hordacre et al. 2021), as well as the establishment of long‐term behavioural habits (Tieges et al. 2015; Wondergem et al. 2022, 2019). Understanding the relationship between walking capacity and movement behaviours during stroke recovery is essential to inform phase‐specific precision rehabilitation. To reduce clinical heterogeneity and allow clearer interpretation of findings, this study focuses specifically on individuals with ischemic stroke, the most common stroke subtype. Therefore, this study aimed to: (1) characterize walking capacity and daily movement behaviours in individuals with acute and subacute ischemic stroke, and (2) explore whether associations between walking capacity and movement behaviours persisted after adjustment for stroke severity.

## 2 Materials and Methods

### 2.1 Study Design and Participants

This cross‐sectional study described and compared walking capacity and movement behaviours during the acute and subacute phases of stroke recovery (Bernhardt et al. 2017). Participants in the acute phase were recruited from an acute stroke hospital, while those in the subacute phase were recruited from a tertiary rehabilitation hospital following transfer from acute care. All participants had returned or were preparing to return to the community at enrolment in the study. The acute care group completed assessments within 7 days of stroke, whereas the subacute group completed assessments between 2 weeks and 3 months post‐stroke. Inclusion criteria were: age ≥ 18 years; ability to stand from a chair and walk 5 m with or without a gait aid; and ability to follow two‐step commands. Exclusion criteria included inability to provide informed consent or follow study instructions, pre‐existing physical disabilities limiting mobility, and medical instability. Cognitive and communication status were assessed as part of routine clinical care; no additional study‐specific cognitive or aphasia screening was conducted for study eligibility.

### 2.2 Procedures

Ethical approval was obtained from the institutional Health Research Ethics Board, and operational approval was granted by the health authorities at both participating sites (Acute: Pro00127990; Subacute: Pro00140017). Eligible participants were identified by physical therapists at participating sites based on the established inclusion criteria. All participants provided written informed consent prior to enrolment. Demographic and stroke‐related data were collected within the specified assessment windows (≤ 7 days for the acute group and 2 weeks to 3 months for the subacute group). In line with recommended measurements for stroke recovery (Kwakkel et al. 2017), stroke location, side affected, and stroke severity at onset, assessed using the National Institutes of Health Stroke Scale (NIHSS; range 0–42) were extracted from patients’ electronic medical records. Higher NIHSS scores represent greater neurological impairment. Health‐related quality of life was assessed using the EuroQol 5‐dimension (EQ‐5D) visual analogue scale (VAS) in the subacute group only (Golicki et al. 2015).

### 2.3 Performance Outcomes

Movement behaviours were objectively measured using an activPAL4 inclinometer and triaxial accelerometer (PAL Technologies, Glasgow, Scotland). The activPAL4, weighing approximately 9g and measuring 26 × 43 × 5 mm, was enclosed in a nitrile sleeve and affixed to the anterior thigh of the non‐hemiparetic leg using a waterproof transparent dressing. The activPAL has been extensively validated in stroke populations, demonstrating high accuracy for detecting posture and postural transitions (Fini et al. 2023; Taraldsen et al. 2011). Participants were instructed to wear the monitor continuously for up to 4 days to capture movement behaviours, as 2 days of measurement provide reliable estimates of physical activity after stroke (Fini et al. 2019). Data were processed using the PAL analysis software (Version 9). The raw data were downloaded and summarized into epoch files (Edwardson et al. 2017). We used PAL’s CREA algorithm to process activPAL data and classify movement behaviours, including primary lying time, sedentary time, standing time, stepping time, daily step count, and sit‐to‐stand transitions. Primary lying time was identified by the CREA algorithm as the longest non‐upright period within each 24‐h period. Waking periods were derived from activPAL event files using the noon‐to‐noon protocol described by Winkler et al. (2016) and confirmed through visual inspection. The percentage of waking time spent sedentary was calculated as total sedentary time divided by total waking time. Only participants with at least one valid day of activPAL recording, defined as 24 hours of continuous wear, were included in the analyses.

### 2.4 Walking Capacity Outcomes

Walking capacity was evaluated using three standardized clinical measures conducted by trained research personnel. Walking endurance was assessed using the 6‐Minute Walk Test (6MWT; Fulk et al. 2008), where participants were instructed to walk as far as possible at a comfortable pace for 6 minutes. Resting periods were permitted if necessary, and the total distance covered was recorded in metres. Functional mobility and dynamic balance were measured using the Timed Up and Go (TUG) test, which required participants to rise from a standard armchair, walk a distance of 3 m, turn around, return to the chair, and sit down (Podsiadlo and Richardson 1991). The time taken to complete the entire task was recorded in seconds using a stopwatch. Lastly, gait speed was assessed using a 10‐m walk test (Moore et al. 2018). Participants were asked to walk a total of 10 m at their usual, self‐selected pace, and the time taken to cross the central 6 m was recorded to eliminate the effects of acceleration and deceleration. The average of three measurements was calculated. Gait speed was expressed in metres per second. Participants were further classified into functional ambulation categories based on comfortable gait speed cut‐offs: household ambulators (< 0.4 m/s), limited community ambulators (0.4–0.8 m/s), and full community ambulators (≥ 0.8 m/s) (Perry et al. 1995).

### 2.5 Statistical Analysis

Statistical analyses were performed using STATA 14. Normality was assessed using the Shapiro‐Wilk test and visual inspection of histograms. Continuous variables are presented as mean (SD) when normally distributed in both groups and as median [interquartile range, IQR] otherwise, while categorical variables are reported as frequencies and percentages. Between‐group comparisons (acute vs. subacute) were performed using Welch’s *t*‐tests for normally distributed variables, Mann‐Whitney U tests for non‐normally distributed variables, and chi‐square or Fisher’s exact tests for categorical variables. Outcomes included movement behaviours (sedentary time, primary lying time, standing time, stepping time, number of bouts, sit‐to‐stand transitions, and daily step count) and walking capacity measures (6MWT, gait speed, and TUG). Associations between demographic and clinical factors (e.g., age, NIHSS), walking capacity, and movement behaviours were examined using Spearman’s rank‐order correlations. Correlation strength was interpreted as weak (0.10–0.39), moderate (0.40–0.69), or strong (≥ 0.70) (Cohen 2013). Statistical significance was set at *p* < 0.05. Exploratory partial Spearman correlations adjusted for NIHSS were subsequently performed only for associations that were statistically significant in unadjusted analyses to determine whether relationships persisted after accounting for stroke severity. Given the small sample size, multiple comparisons, and exploratory nature of these analyses, adjusted correlations were considered hypothesis‐generating. No correction for multiple comparisons was applied, and no a priori sample size calculation was performed. All eligible participants recruited during the study period were included.

## 3 Results

A total of 44 participants with ischemic stroke were included (acute: *n* = 19; subacute: *n* = 25). Baseline demographic and clinical characteristics are presented in Table 1. The acute and subacute groups were generally similar in demographics; however, stroke severity differed, with lower NIHSS scores in the acute group than in the subacute group (median NIHSS: 2 [0–4] vs. 7 [4–8]; *p* = 0.002). Time since stroke onset also differed by design (5 [4–5] vs. 51 [32–69] days). Health‐related quality of life, assessed using the EQ‐5D VAS, was reported only for the subacute group, with a mean score of 64.4 ± 13.1.

**TABLE 1:** Demographic and clinical characteristics of the participants.

Characteristic	Total (*n* = 44)	Acute (*n* = 19)	Subacute (*n* = 25)	*p*‐value
Demographics				
Age (years), mean (SD)	67.3 (12.1)	66.3 (12.7)	68.0 (11.9)	0.646
Sex (male), *n* (%)	25 (56.8%)	13 (68.4%)	12 (48.0%)	0.227
Ethnicity, *n* (%)				
Caucasian	34 (77.3%)	14 (73.7%)	20 (80.0%)	0.877
Black	2 (4.5%)	1 (5.3%)	1 (4.0%)	
South Asian	3 (6.8%)	1 (5.3%)	2 (8.0%)	
East Asian	3 (6.8%)	2 (10.5%)	1 (4.0%)	
Indigenous	1 (2.3%)	0 (0.0%)	1 (4.0%)	
Latin American	1 (2.3%)	1 (5.3%)	0 (0.0%)	
Marital status, *n* (%)				
Never married	2 (4.5%)	1 (5.5%)	1 (4.0%)	0.979
Married	29 (65.9%)	13 (68.4%)	16 (64.0%)	
Separated	5 (11.4%)	2 (10.5%)	3 (12.0%)	
Widowed	8 (18.2%)	3 (15.8%)	5 (20.0%)	
Support, *n* (%)				
Lives alone	10 (22.7%)	5 (26.3%)	5 (20.0%)	0.370
With spouse	28 (63.6%)	11 (57.9%)	17 (68.0%)	
Assisted living	2 (4.5%)	2 (10.5%)	0 (0.0%)	
Others (e.g. children)	4 (9.1%)	1 (5.3%)	3 (12.0%)	
Clinical characteristics				
BMI (kg/m^2^), mean (SD)	27.2 (4.6)	28.0 (5.5)	26.6 (3.7)	0.257
Onset days, median (IQR)	32 (5, 59)	5 (4, 5)	51 (32, 69)	—
NIHSS Score, median (IQR)	4.5 (2, 8)	2 (0, 4)	7 (4, 8)	0.002
Systolic BP (mmHg), mean (SD)	129.0 (15.4)	134.3 (15.6)	125.0 (14.2)	0.063
Diastolic BP (mmHg), mean (SD)	77.3 (10.9)	79.1 (11.4)	76.0 (10.4)	0.380
Heart rate (bpm), mean (SD)	72.2 (14.0)	67.4 (13.9)	75.8 (13.2)	0.070
Limbs affected, *n* (%)				
Left	18 (40.9%)	6 (31.6%)	12 (48.0%)	0.322
Right	26 (59.1%)	13 (68.4%)	13 (52.0%)	
Stroke location, *n* (%)				
Cortical	29 (65.9%)	12 (63.2%)	17 (68.0%)	0.681
Subcortical	11 (25.0%)	5 (26.3%)	6 (24.0%)	
Multiple (> 1)	2 (4.5%)	1 (5.3%)	1 (4.0%)	
Unknown	1 (2.3%)	0 (0.0%)	1 (4.0%)	
Cerebellar	1 (2.3%)	1 (5.3%)	0 (0.0%)	
Gait speed categories				
Household (< 0.4 m/s)	3 (6.8%)	2 (10.5%)	1 (4.0%)	0.126
Limited community (0.4–0.8 m/s)	15 (34.1%)	9 (47.4%)	6 (24.0%)	
Community (≥ 0.8 m/s)	26 (59.1%)	8 (42.1%)	18 (72.0%)	

Abbreviations: BMI, Body mass Index; IQR, Interquartile range; NIHSS, National Institute Health Stroke Scale; SD, Standard deviation.

Daily movement behaviours for the acute and subacute groups are presented in Figure 1A–D. There were no statistically significant between‐group differences in primary lying, sedentary, standing, or stepping time (all *p* > 0.05). Despite being ambulatory and able to complete standardized walking capacity assessments, participants in both groups accumulated high levels of sedentary time, exceeding the ≤ 8 hours/day sedentary time threshold recommended in the Canadian 24‐Hour Movement Guidelines for Adults and Older Adults, with the acute group accumulating 702.4 ± 119.5 minutes/day (11.7 hours; 80.9% of waking time) and the subacute group 720.8 ± 106.9 minutes/day (12.0 hours; 77.2% of waking time); *p* = 0.72. Stepping time was not significantly different between groups (median 32.6 [14.6–69.4] vs. 54.0 [36.0–66.0] minutes/day in the acute and subacute groups, respectively; *p* = 0.09), nor was standing time (106.0 [46.0–196.4] vs. 131.0 [97.0–200.0] minutes/day; *p* = 0.17) or primary lying time (541.0 [361.5–565.9] vs. 505.0 [428.0–582.0] minutes/day; *p* = 0.61).

**FIGURE 1:**
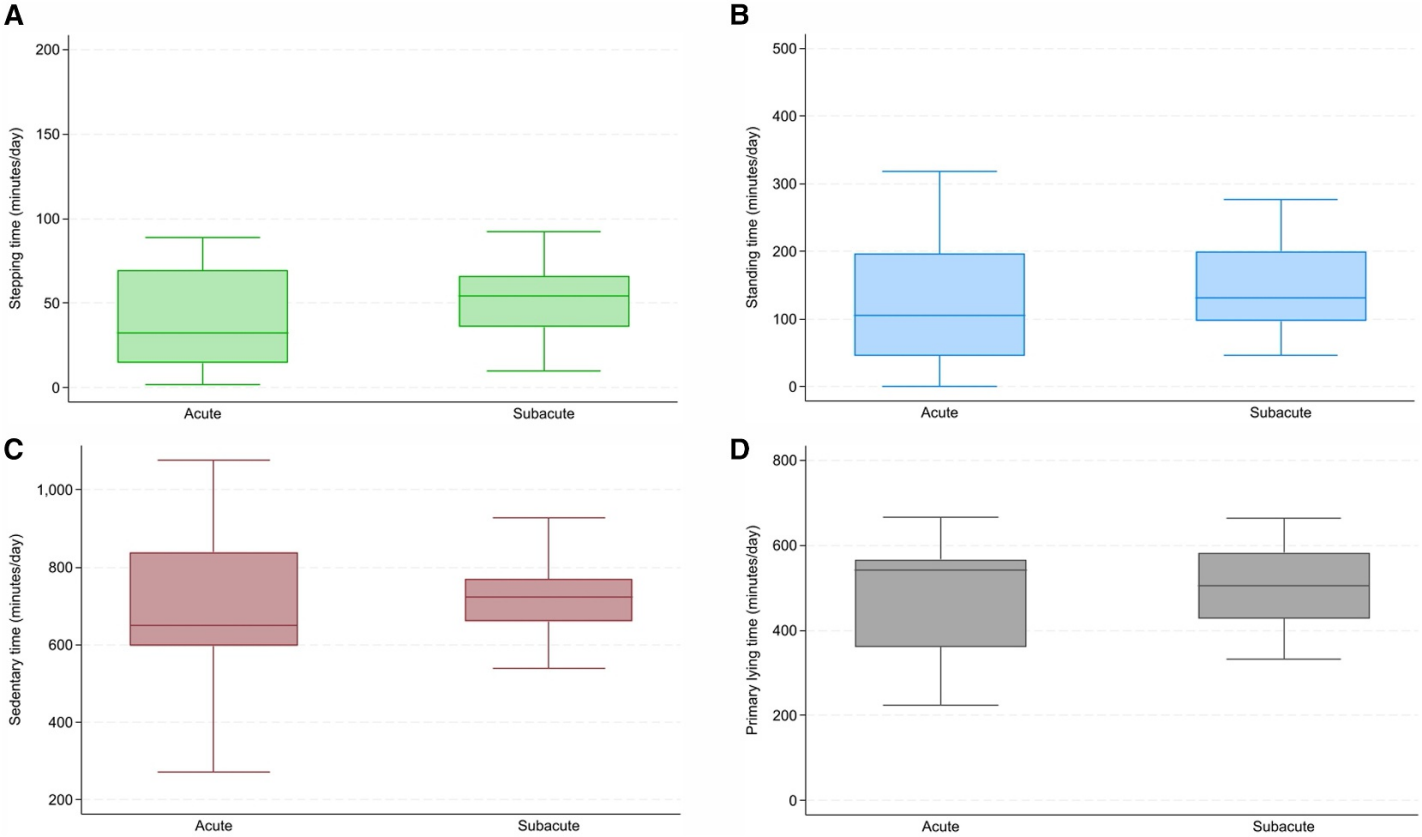
(A–D) Box plots of 24‐h movement behaviours (stepping, standing, sedentary, and primary lying) in acute and subacute phases of ischemic stroke.

The patterns of sedentary behaviour accumulation, sit‐to‐stand transitions, and daily step count are presented in Table 2. There were no statistically significant differences between the groups in sedentary bout frequency, sit‐to‐stand transitions, or daily step count (all *p* > 0.05). In both groups, most sedentary bouts lasted less than 30 minutes (median 28.5 [10.0–47.0] vs. 32.0 [28.0–43.0] bouts/day; *p* = 0.21), followed by bouts of 30–60 minutes (3.5 [2.0–5.0] vs. 4.0 [3.8–5.0]; *p* = 0.15), while very prolonged sedentary bouts (> 4 hours) were rare (median 0 in both groups). The number of sit‐to‐stand transitions per day was comparable between groups (38 [19–56] vs. 42 [39–53]; *p* = 0.20) as was the daily step count (2575 [835–4718] vs. 3494 [2118–5213] steps/day; *p* = 0.09).

**TABLE 2:** Comparison of sedentary bout frequencies, sit‐to‐stand transitions and step count.

Characteristic	Acute (*n* = 19)	Subacute (*n* = 25)	*p*‐value
Sedentary bout frequency (*n*/day)			
Bouts < 30 minutes	28.5 [10.0–47.0]	32.0 [28.0–43.0]	0.21
Bouts 30 minutes–1 hour	3.5 [2.0–5.0]	4.0 [3.8–5.0]	0.15
Bouts 1 hour–2 hours	2.0 [1.0–3.0]	2.0 [2.0–3.0]	0.83
Bouts 2–4 hours	1.0 [0.0–1.0]	1.0 [0.3–1.0]	0.24
Bouts > 4 hours	0.0 [0.0–0.0]	0.0 [0.0–0.0]	0.75
Sit‐to‐stand transitions (*n*)	38 [19–56]	42 [39–53]	0.20
Daily step count (*n*)	2575 [835–4718]	3494 [2118–5213]	0.09

*Note:* Values are median [interquartile range].

Abbreviation: IQR, interquartile range.

Walking capacity outcomes are presented in Table 3. The subacute group walked faster than the acute group (0.89 ± 0.24 vs. 0.71 ± 0.28 m/s; *p* = 0.03) and completed the TUG in a shorter time (median 15.9 [11.0–22.3] vs. 19.0 [17.0–24.8] seconds; *p* = 0.03), whereas 6MWT distance did not differ significantly between groups (314.2 ± 104.8 vs. 283.1 ± 76.1 m; *p* = 0.26). Correlations between demographic factors, clinical characteristics, walking capacity, and movement behaviours are presented in Tables S1–S3. Gait speed was the only walking capacity measure consistently associated with movement behaviours in this sample. In the acute group, faster gait speed was associated with lower sedentary time and greater stepping time, standing time, daily step count, and sit‐to‐stand transitions (Table S1). Similar associations were observed in the subacute group (Table S2). In contrast, 6MWT and TUG were not significantly associated with movement behaviours in either group. Older age was associated with greater sedentary time in the acute group (*ρ* = 0.51, *p* < 0.05).

**TABLE 3:** Comparison of walking capacity measures.

Characteristic	Acute (*n* = 19)	Subacute (*n* = 25)	Mean difference (95% CI)	*p*‐value
Walking capacity				
6MWT (metres)	283.1 (76.1)	314.2 (104.8)	−31.1 (−86.0 to 23.9)	0.26
Gait speed (m/s)	0.71 (0.28)	0.89 (0.24)	−0.18 (−0.33 to −0.02)	0.03*
TUG (seconds)^a^	19.0 [17.0–24.8]	15.9 [11.0–22.3]	—	0.03*

*Note:* Values are mean (SD), unless otherwise indicated.

Abbreviations: 6MWT, 6‐Minute Walk Test; CI, confidence interval; SD, standard deviation; TUG, Timed Up and Go.

^a^ Median [interquartile range].

^*^ Significant at *p* < 0.05.

Given differences in stroke severity between groups and the exploratory nature of these analyses, partial Spearman correlations adjusted for NIHSS were performed to examine whether associations between gait speed and movement behaviours persisted after accounting for stroke severity (Table S3). After adjustment, the association between gait speed and sedentary time remained significant in both the acute (r_partial = −0.65, *p* < 0.01) and subacute groups (r_partial = −0.49, *p* < 0.05). In the subacute group, gait speed remained positively associated with daily step count (r_partial = 0.61, *p* < 0.01), stepping time (r_partial = 0.52, *p* < 0.01), and standing time (r_partial = 0.49, *p* < 0.05). In the acute group, associations with daily step count, stepping time, standing time, and sit‐to‐stand transitions were attenuated after adjustment and were no longer statistically significant. Primary lying time was not associated with gait speed in either group.

In the subacute group, quality of life (EQ‐5D VAS) was not significantly associated with walking capacity measures or movement behaviours. However, higher stroke severity (NIHSS) and longer time since stroke onset were associated with lower EQ‐5D VAS scores (*ρ* = −0.57, *p* < 0.01 and *ρ* = −0.41, *p* < 0.05, respectively).

## 4 Discussion

This study characterized walking capacity and daily movement behaviours in two separate groups of adults in the acute and subacute phases of ischemic stroke recovery. Although the subacute group had higher gait speed and lower TUG scores than the acute group, daily movement behaviours were broadly similar between groups, with both groups accumulating approximately 12 hours of sedentary time per day. Gait speed was the only walking capacity measure most consistently associated with daily movement behaviours, with faster gait speed associated with lower sedentary time and greater stepping and standing time. In exploratory analyses adjusted for stroke severity, the association between gait speed and sedentary time remained significant in both groups, whereas associations with stepping, standing, and daily step count remained significant only in the subacute group. These findings are consistent with a potential capacity‐performance gap during early stroke recovery (Lang et al. 2023; Lundquist et al. 2022; Thompson et al. 2024; Waddell et al. 2017). However, because the acute and subacute groups were recruited independently, this study cannot determine whether improvements in walking capacity translate into improvements in daily movement behaviours within individuals.

The accumulation of approximately 12 hours of daily sedentary time is the most clinically important finding of this study, particularly given that participants were ambulatory and able to complete standardized walking capacity assessments. This is consistent with previous reports of high sedentary time during and after inpatient stroke rehabilitation and early stroke recovery (Barrett et al. 2018; Ezeugwu and Manns 2017; Mattlage et al. 2015). Importantly, this level of sedentary time exceeds the ≤ 8 hours/day threshold recommended by the Canadian 24‐Hour Movement Guidelines for Adults and Older Adults (Ross et al. 2020). A meta‐analysis of 19 studies found that adults in the highest sedentary time category (median: 10.5 hours/day) had a 24% higher risk of cardiovascular disease morbidity and a 29% higher risk of cardiovascular disease mortality compared with those in the lowest category (median: 2.8–3.0 hours/day) (Jingjie et al. 2022). Sedentary time > 9 hours/day has also been associated with an increased risk of future cardiovascular disease (Ajufo et al. 2025; Pandey et al. 2016). Although prolonged sedentary bouts exceeding 4 hours were uncommon in our study, the overall volume remained high, suggesting that physical therapists should consider strategies to reduce total sedentary time.

Previous research suggests that the extent to which walking capacity translates into daily movement behaviours may be influenced by behavioural, environmental, and contextual factors, such as fear of falling, perceptions that walking practice during structured therapy is sufficient, and environmental or social barriers that limit opportunities for walking (Lang et al. 2023; Kenah et al. 2024). Qualitative studies suggest that falls‐prevention policies, overprotective staff attitudes, and highly structured hospital environments may limit self‐directed activity outside formal therapy sessions (Kenah et al. 2024), while modifying the rehabilitation environment may increase physical activity levels (Rosbergen et al. 2017). A recent meta‐analysis also suggests that exercise‐only interventions are associated with higher walking capacity measures, while behaviour change techniques (e.g., goal setting, self‐monitoring, feedback) are more strongly associated with daily movement behaviours (Ezeugwa et al. 2026).

Taken together, the findings from this study suggest that stroke rehabilitation should consider daily movement behaviours in addition to walking capacity. Standardized walking assessments provide valuable information about functional capacity but may not fully reflect how much patients move throughout the day. Gait speed was the only walking capacity measure associated with daily movement behaviours in this study. In exploratory analyses adjusted for stroke severity, gait speed remained associated with sedentary time in both groups, whereas associations with stepping and standing time were attenuated in the acute group. Given the sample size and exploratory design, this should not be interpreted as evidence that gait speed is a superior measure; rather, it reinforces that standardized walking capacity measures and daily movement behaviours provide complementary information. The considerable inter‐individual variability in daily step count further highlights that individuals with similar levels of walking capacity may demonstrate different daily movement behaviours. Behaviour‐focused strategies incorporating goal setting, self‐monitoring, feedback, and activity monitoring may represent potential approaches to address this potential gap between walking capacity and daily movement behaviours (Ashizawa et al. 2022; Ezeugwu and Manns 2018; Thompson et al. 2024).

A key strength of this study was the objective assessment of daily movement behaviours using activPAL alongside standardized walking‐capacity measures, allowing these related but distinct constructs to be examined concurrently. Exploratory NIHSS‐adjusted analyses provided additional context for the associations between gait speed and daily movement behaviours, although these findings should be interpreted cautiously.

### 4.1 Limitations

This study has several limitations. First, its cross‐sectional design precludes causal inference and does not allow assessment of individual recovery trajectories over time. Second, no a priori sample size calculation was performed, and the sample was limited to eligible participants recruited during the study period, which may have reduced the power to detect smaller between‐group differences or weaker associations. Third, analyses were not adjusted for multiple comparisons, and NIHSS‐adjusted analyses were performed only for associations that were significant in unadjusted analyses; therefore, findings should be considered exploratory and hypothesis‐generating. Fourth, acute and subacute groups were recruited from different care settings and represented distinct clinical pathways rather than longitudinal stages of recovery within the same individuals. Consequently, the findings cannot demonstrate whether gains in walking capacity fail to translate into gains in daily movement behaviours within individuals. Stroke severity differed between groups, and although analyses examining the association between gait speed and movement behaviours were adjusted for NIHSS, residual confounding cannot be excluded. Lastly, the sample included ambulatory individuals with relatively mild neurological impairment who were discharged home; therefore, findings should not be generalized to the broader acute or subacute stroke population, including non‐ambulatory individuals or those requiring long‐term institutional care.

## 5 Conclusion

In this ambulatory sample of individuals in the acute and subacute phases of ischemic stroke recovery, participants accumulated substantial sedentary time, with daily movement behaviours broadly similar between groups. Gait speed was the only walking capacity measure consistently associated with daily movement behaviours. These findings are consistent with a potential capacity‐performance gap during early stroke recovery and underscore the importance of considering daily movement behaviours alongside standardized walking capacity measures.

### 5.1 Implications for Physiotherapy Practice

- High sedentary time in both acute and subacute phases highlights the importance of considering movement behaviours early after stroke.
- Assessment of walking capacity alone may not capture daily movement behaviours.
- Physiotherapists may consider assessing and addressing daily movement behaviours during inpatient rehabilitation and the transition from structured hospital care to home.

## Author Contributions

All authors contributed to the project and met the International Committee of Medical Journal Editors (ICMJE) authorship criteria. J.C.E. conceptualised the study. J.C.E., A.S.‐R., and M.I. were involved in data collection. J.C.E., and V.E.E. processed and analysed the wearable sensor data. J.C.E. drafted the manuscript and all authors critically reviewed the manuscript and approved the final version. V.E.E. secured funding.

## Funding

This study received funding support from the Glenrose Hospital Foundation. JCE received personnel salary awards from the Heart and Stroke Foundation of Canada (HSFC), Brain Canada, the Canadian Consortium of Clinical Trial Training (CANTRAIN) and Alberta Innovates. VEE received the Heart and Stroke Foundation of Canada (HSFC) National New Investigator Award.

## Ethics Statement

Ethics approvals were granted by the University of Alberta’s Health Research Ethics Board (HREB) (Acute: Pro00127990 [February 3, 2023]; Subacute: Pro00140017 [March 12, 2024]) and all participants provided written informed consent.

## Conflicts of Interest

The authors declare no conflicts of interest.

## Supporting information


Supporting Information S1

## Data Availability

De‐identified participant data will be available upon reasonable request from the corresponding author following publication, subject to approval by regulatory authorities.
