# Supplementary material for: Walking Capacity and Daily Movement Behaviours After Ischemic Stroke: A Potential Capacity‐Performance Gap
Source: Physiother Res Int. 2026 Nov 3;32(1):e70372. doi: 10.1002/pri.70372 (PMC13730178; doi:10.1002/pri.70372)
Supplement: Supplementary file 1 [file PRI-32-e70372-s001.docx]

**Table S1: Correlations between walking outcomes and demographic, and clinical variables – Acute Phase (n=19)**

| **Variable** | **Outcome Measure** | **Age** | **BMI** | **NIHSS** | **Onset** | **10MWT** | **6-MWT** | **TUG** |
| --- | --- | --- | --- | --- | --- | --- | --- | --- |
| **Movement behaviours** | Primary lying (min) | -0.34 | -0.19 | -0.06 | -0.33 | 0.34 | 0.41 | -0.33 |
|  | Sedentary (min) | 0.51* | 0.06 | 0.08 | 0.02 | -0.68** | -0.26 | 0.32 |
|  | Standing (min) | -0.29 | -0.08 | 0.14 | 0.01 | 0.51* | 0.15 | -0.18 |
|  | Stepping (min) | -0.43 | 0.20 | 0.21 | 0.14 | 0.56* | 0.30 | -0.32 |
|  | Steps per day (n) | -0.45 | 0.16 | 0.20 | 0.17 | 0.61** | 0.37 | -0.37 |
|  | Sit-to-Stand (n) | -0.26 | 0.25 | 0.33 | -0.03 | 0.55* | 0.12 | -0.24 |
| **Demographic/Clinical** | Age | 1.00 | 0.00 | 0.21 | 0.06 | -0.34 | -0.37 | 0.33 |
|  | BMI | 0.00 | 1.00 | 0.15 | 0.03 | 0.02 | 0.15 | 0.04 |
|  | NIHSS | 0.21 | 0.15 | 1.00 | -0.07 | 0.13 | -0.26 | 0.12 |
|  | Onset | 0.06 | 0.03 | -0.07 | 1.00 | -0.12 | -0.18 | 0.47 |

Abbreviations: TUG = Timed Up and Go; 10MWT = 10 metre walk test (gait speed); 6-MWT = 6minute walk test (walking endurance).

*Correlation significant at p less than 0.05

**Correlation significant at p less than 0.01

**Table S2: Correlations between walking outcomes and demographic, clinical, and quality of life variables – Subacute Phase (n=25)**

| **Variable** | **Outcome Measure** | **Age** | **BMI** | **NIHSS** | **Onset** | **10MWT** | **6-MWT** | **TUG** | **EQ-5D** |
| --- | --- | --- | --- | --- | --- | --- | --- | --- | --- |
| **Movement behaviour** | Primary lying(min) | -0.19 | -0.36 | -0.14 | 0.02 | 0.01 | 0.17 | -0.08 | 0.32 |
|  | Sedentary (min) | -0.00 | 0.31 | -0.11 | -0.27 | -0.41* | -0.36 | 0.19 | 0.08 |
|  | Standing (min) | 0.30 | 0.20 | 0.12 | 0.31 | 0.43* | 0.24 | -0.15 | -0.26 |
|  | Stepping (min) | -0.00 | -0.09 | 0.20 | 0.06 | 0.40* | 0.19 | -0.32 | -0.17 |
|  | Steps per day (n) | -0.01 | -0.07 | 0.19 | 0.15 | 0.50** | 0.34 | -0.36 | -0.19 |
|  | Sit-to-Stand (n) | 0.26 | -0.16 | -0.17 | 0.00 | -0.01 | -0.02 | -0.15 | 0.35 |
| **Demographic/Clinical** | Age | 1.00 | 0.21 | -0.17 | -0.14 | -0.02 | -0.35 | 0.21 | 0.34 |
|  | BMI | 0.21 | 1.00 | -0.16 | -0.02 | 0.07 | -0.18 | -0.13 | 0.03 |
|  | NIHSS | -0.17 | -0.16 | 1.00 | 0.04 | -0.29 | -0.13 | 0.34 | -0.57** |
|  | Onset | -0.14 | -0.02 | 0.04 | 1.00 | 0.46* | 0.52** | -0.21 | -0.41* |

Abbreviations: TUG = Timed Up and Go; 10MWT = 10 metre walk test (gait speed); 6-MWT = 6minute walk test (walking endurance); EQ-5D (VAS) = EuroQol 5-Dimension Visual Analogue Scale

*Correlations significant at p < 0.05

**Correlations significant at p < 0.01

**Table S3: Correlations Between Movement Behaviours and Gait Speed.**

| **Variable** | **Acute (**$n=19$**)** |  | **Subacute (**$n=25$**)** |  |
| --- | --- | --- | --- | --- |
| **Movement Behaviour** | **Unadjusted (**$\rho$**)** | **Adjusted (**$r_{partial}$**)** | **Unadjusted (**$\rho$**)** | **Adjusted (**$r_{partial}$**)** |
| Sedentary (min) | -0.68** | -0.65** | -0.41* | -0.49* |
| Steps per day (n) | 0.61** | 0.43 | 0.50**** | 0.61** |
| Stepping (min) | 0.56* | 0.40 | 0.40* | 0.52** |
| Standing (min) | 0.51* | 0.40 | 0.43* | 0.49* |
| Sit-to-Stand (n) | 0.55* | 0.45 | -0.01 | -0.02 |
| Primary lying (min) | 0.34 | 0.31 | 0.01 | -0.01 |

**Note:** Unadjusted coefficients are Spearman’s rank correlations ($\rho$). Adjusted coefficients are partial rank correlations ($r_{partial}$), controlling for stroke severity (NIHSS).

*Correlations significant at p < 0.05

**Correlations significant at p < 0.01
